# Genetic and Physical Localization of the Gene Controlling Leaf Pigmentation Pattern in *Medicago truncatula*

**DOI:** 10.1534/g3.120.401689

**Published:** 2020-09-10

**Authors:** Xiaocheng Yu, Qiulin Qin, Xia Wu, Dandan Li, Shengming Yang

**Affiliations:** Department of Plant & Soil Sciences, University of Kentucky, Lexington, KY 40546

**Keywords:** Anthocyanin biosynthesis, *M. truncatula*, Genetic mapping, leaf pigmentation

## Abstract

In *Medicago truncatula*, some ecotypes form a black or purple stain in the middle of adaxial leaf surface due to accumulation of anthocyanins. However, this morphological marker is missing in some other ecotypes, although anthocyanin biosynthesis pathway is not disrupted. Genetic analysis indicated that the lack of the leaf spot of anthocyanins accumulation is a dominant trait, which is controlled by a single gene, *LPP1*. Genetic mapping indicated that the *LPP1* gene was delimited to a 280 kb-region on Chromosome 7. A total of 8 protein-coding genes were identified in the *LPP1* locus through gene annotation and sequence analysis. Of those, two genes, putatively encoding MYB-transcriptional suppressors, were selected as candidates for functional validation.

*Medicago truncatula* is a model legume plant closely related to alfalfa (*Medicago sativa*), one of the most important forage crops worldwide. Due to its diploidy, small and deeply sequenced genome, abundance of natural variation and efficacy of gene transformation, *M. truncatula* has been widely used for genomic studies that are legume-specific, such as gene discovery in nodule symbiosis signaling (Cook 1999). The genome data of *M. truncatula* has also been translated to alfalfa improvement in disease resistance, forage quality, biomass yield and abiotic stress tolerance (Yang *et al.* 2008; Yang *et al.* 2013; Peng *et al.* 2018; Zhou *et al.* 2011b). In addition, *M. truncatula* has been a subject for studies of molecular mechanisms underlying organogenesis of leaf, flower, seed and root tissues (Franssen *et al.* 2015; Zhou *et al.* 2011a; Le Signor *et al.* 2018; Cheng *et al.* 2018). Some unique morphological traits, such as leaf pigmentation and pod helical coiling, make *M. truncatula* a special model plant for developmental studies.

Particularly at the seedling stage, plants of some *M. truncatula* accessions are characterized by a black or purple stain in the middle of the adaxial leaf surface, which results from the accumulation of anthocyanins. Among the flavonoids, anthocyanins are water-soluble vacuole pigments with strong antioxidant activities. Not only in leaves, anthocyanins are also accumulated in the stem, flower and seed coat. They can provide plants with bright flower colors for attracting pollinators and offer protection from microbial pathogens, insects and high light/UV-damage (Mouradov and Spangenberg 2014; Koes *et al.* 2005). Anthocyanins have also been recognized for their beneficial health effects on human chronic diseases due to their strong antioxidant activities (Khurana *et al.* 2013).

In *Arabidopsis*, anthocyanins and proanthocyanidins (PAs; also known as condensed tannins) share early steps in their biosynthetic pathways, but diverge after formation of anthocyanidin, the precursor of both anthocyanins and PAs. Expression of enzymes involved in anthocyanin and PA biosynthesis is regulated by MBW, a complex of transcription factors composed of R2R3-MYB, basic helix-loop-helix (bHLH), and WD40 repeat proteins(Gonzalez *et al.* 2008). The functional orthologs of the MBW complex components have been identified in *M. truncatula*. One R2R3-MYB protein known to regulate only anthocyanin biosynthesis in *M. truncatula* is MtLAP1 (Peel *et al.* 2009), while three (MtPAR, MtMYB14, and MtMYB5) have been shown to be involved in PA biosynthesis (Verdier *et al.* 2012; Liu *et al.* 2014). Screening of mutants with altered pigmentation patterns introduced by Tnt1 retrotransposon insertion led to the cloning of *MtWD40-1* and *MtTT8* (a bHLH gene), which are involved in both the PA and anthocyanin pathways (Pang *et al.* 2009; Li *et al.* 2016).

Interestingly, although disruption of MBW or other genes results in the disappearance of leaf pigmentation in *M. truncatula* accessions which have this morphological marker (Pang *et al.* 2009; Li *et al.* 2016; Carletti *et al.* 2013), genetic analysis with natural variation indicates that the lack of the leaf spot from anthocyanin accumulation is a dominant trait (Penmetsa and Cook 2000). The absence of this leaf marking is controlled by a single gene, which has not been identified or characterized yet.

In the present study, we finely mapped the gene regulating accumulation of anthocyanins in leaves (namely *LPP1* for *Leaf Pigmentation of Anthocyanins 1*). The *LPP1* gene was located on the chromosome 7. Sequence analysis and gene annotation enabled selection two MYB-transcription factor genes as candidates of *LPP1*.

## Materials and methods

### The mapping population

The *M. truncatula* genotype Jemalong A17 (A17 hereafter) displays the leaf marking, whereas A20 and F3005.5 (F83005 hereafter) exbibits the opposite phenotype on leaves. Three different segregating populations were used for genetic mapping of the *LPP1* gene. Of those, 129 recombinant inbred lines (RILs) and 269 F_2_s were derived from a cross between the *M. truncatula* genotypes A17 and A20, and 203 F_2_s were derived from A17 × F83005. The phenotype of F_2_ recombinants were confirmed with at least 30 F_3_ plants. Seedlings of parents and the segregating populations were grown in a growth room with a 16 h light, 23°/8 h dark, 20° regime.

### Phenotyping of leaf pigmentation

Leaf pigmentation pattern was visually determined three weeks after seed germination, which was double confirmed one week afterward.

### Marker development and genetic mapping

CAPS (cleaved amplified polymorphic sequences) markers were developed based on SNPs (single nucleotide polymorphisms) identified between the two parents (Li *et al.* 2014). DNA sequencing-PCR was conducted with the Dye Terminator Cycle Sequencing (DTCS) Quick Start Kit (Beckman Coulter). After ethanol precipitation and purification, the PCR product was resuspended with 40µl Sample Loading Solution (SLS, Beckman Coulter) before loading into the sequencing instrument (CEQ8000 Genetic Analysis System, Beckman Coulter). The genetic map was constructed using the software JoinMap version 3.0 (You *et al.* 2010). All markers used in this study are given in Table 1.

**Table 1:** Sequences and locations for primers used in genetic mapping of *LPP1*

Marker name	Position	Forward primer	Reverse primer	Restriction enzymes to distinguish A17 and A20 alleles	Restriction enzymes to distinguish A17 and F83005 alleles
M1	12086486...12087011	TCATCAATTCCGTCCAACAA	TATCCCTCGCGTATCCTCAC	Hpy188I	No polymorphism
M2	12109541...12109966	AAATGGGAATGGATGGTTGA	ACTTGCATTGAAGGGGTGAC	AflIII	HinCII
M3	12816505...12816917	GTAGTCGCCCATCATTGGTC	GAAACCGTCCTCGTTGATACA	TaqI	Dominant
M4	12971231...12971706	CCTGTGCGATTGAAGTTGTTT	GCAATATTCCCTGAGCCAAG	HinfI	Hpy188I
M5	13100151...13100643	CAATGGCACAATGGGTAAAA	CGGTGAGGAAAGAAAGTTAGAGA	AvaII	HincII
M6	13170690...13171159	CCTGGCGAGTTTTGTTTTGT	AAAATGCAAATGCAACGACA	Tsp509I	BtgI
M7	13291032...13291523	GAGCTTCCAAATACCCTTTCAA	TGCTAGCTTCCATGTTGTGC	BglII	AflII
M8	13294389...13294915	CTGGCGTAGAGGATCACTGG	TGGGGGTGGTGAGAACTCTA	ApoI	ApoI
M9	13298918...13299373	GTGATGTTCCACTTCAACACG	CATATCATAATAATGGGGGTTGG	PshAI	PshAI
M10	13309086...13309536	AAAGTGGCCACCACAGTAGG	GAGAGGGAGAGGGGAGAATG	AseI	StyI
M11	13487194...13487665	AATGACCACTGGGGGTTAGA	CGGTGATAATGATTTCCTTGC	BclI	No polymorphism
M12	13581669...13582122	CCGAAGCTTGTGGACAATTA	CCACCGGTCACTGTCCTATT	BstYI	BstYI
M13	13606833...13607293	TTGGTTGGAAATAAGTGTCAAGG	CCAAACAAGGGTAAACCAACA	AflIII/	DraI
M14	13714308...13714799	CATCGACCTTGATAGCCACA	AGTGCAGACGCTTACCACAA	HpyCH4V	HpyCH4V
M15	13807044...13807513	GAAAGCCACCGGTCAGAGTA	CCTTGGACGAATAATTGAGACA	Size polymorphism	Size polymorphism
M17	13854196...13854645	AATACCAAATGGAGGGTGGA	ATAAAGGCACAATTGGCAAAC	Size polymorphism	Size polymorphism
M18	13856315...13856782	TGTTCACTTCCAAAGGATTTCA	GGCATGACTATGTGGGACCT	Size polymorphism	Size polymorphism
M20	14304994...14305467	AACCTCTAAACGGCCAAGGT	GTGCAGGAGCATCAGACAAA	Size polymorphism	Size polymorphism
M21	14552501...14552956	CGCAGAGGATACCCGTTTAC	CAAGTTGGGAAAATAGGGTGTT	No polymorphism	BsaBI

### Genomic PCR analysis

Genomic DNA was isolated using the CTAB method and used for PCR with Taq DNA polymerase (New England Biolab). The thermal amplification program was as follows: denaturation at 95° for 2 min, followed by 35 cycles of 94° for 30 s, 55° for 30 s, and 72° for 60 s, with a final extension at 72° for 5 min.

### Physical mapping and sequence analysis

The genome sequence of *M. truncatula* Mt4.0 was used for marker identification and physical mapping (Tang *et al.* 2014). Gene prediction and annotation provided by the *M. truncatula* genome database (http://www.medicagogenome.org) was confirmed with the programs FGENESH and Pfam 32.0, respectively (Bateman *et al.* 2004; Solovyev and Salamov 1997).

### Phylogenetic analysis

Full-length protein sequences of 26 Myb transcription factors involved in anthocyanin biosynthesis and the allelic coding products of candidate genes were aligned using the Clustal X program (Larkin *et al.* 2007) (Table S1). NJplot was used to construct the phylogenetic tree. The bootstrap consensus tree inferred from 1,000 replicates was taken to represent the similarity of the analyzed protein sequences. Evolutionary distances were computed using the Maximum Composite Likelihood method (Tamura *et al.* 2011) and are presented as the number of amino acid (aa) substitutions per site.

### Real-time PCR (qRT-PCR) analysis

RNA isolated from young leaves and flowers of *M. truncatula* plants was used for qRT-PCR analysis for the candidate genes with four replicates, and each biological replicate consisted of tissues from at least 5 plants. Young leaves were sampled from seedlings three weeks after gemination. Open flowers were collected when they were in full bloom. Total RNA was extracted using the RNeasy Plant Mini Kit (Qiagen, Valencia, CA, USA). First-strand cDNA was synthesized using M-MLV Reverse Transcriptase (Invitrogen, Carlsbad, CA, USA) according to the manufacturer’s instructions. Fluorescent PCR amplifications were performed in triplicate using the StepOne real-time PCR system (Applied Biosystems, Grand Island, NY, USA). Aliquots of each first strand cDNA (2 µL), equivalent to 20 ng of total RNA, were used for PCR amplification in 20 µL reactions containing 2 μL of each gene-specific primer (2.5 μM), 8.8 μL of water, and 10 μL of iTaq SYBR Green Supermix with ROX (Bio-Rad, Hercules, CA, USA). The *actin* gene was used as the internal control for real-time analysis and was amplified with forward (5′- TCAATGTGCCTGCCATGTATGT-3′) and reverse (5′- ACTCACACCGTCACCAGAATCC-3′) primers. Primers for candidate genes were given in Table S2. Amplification conditions were as follows: denaturation at 95° for 2 min, followed by 35 cycles of 95° for 30 s, 51° for 30 s, and 72° for 30 s, with a final extension at 72° for 5 min.

### Data availability

All data are included in the paper, tables, figures or the associated supplemental materials. Figure S1 presented a phylogenetic tree based on the allelic products of the *LPP1* candidate genes. Domain structure analysis of the Myb proteins was shown in Figure S2. Sequence information for the Myb proteins used for the phylogenetic analysis could be found in Table S1. Table S2 showed the primer sequences used in the present study. Tables S3, S4 and S5 included comparation of allelic sequences for the *LPP1* candidate genes. The *M. truncatula* genome sequence and gene annotation are available from public repositories (http://www.medicagogenome.org). All other reagents are commercially available or can be sent upon request. Supplemental material available at figshare: https://doi.org/10.6084/m9.figshare.12846581.

## Results and Discussion

**Figure 1:**
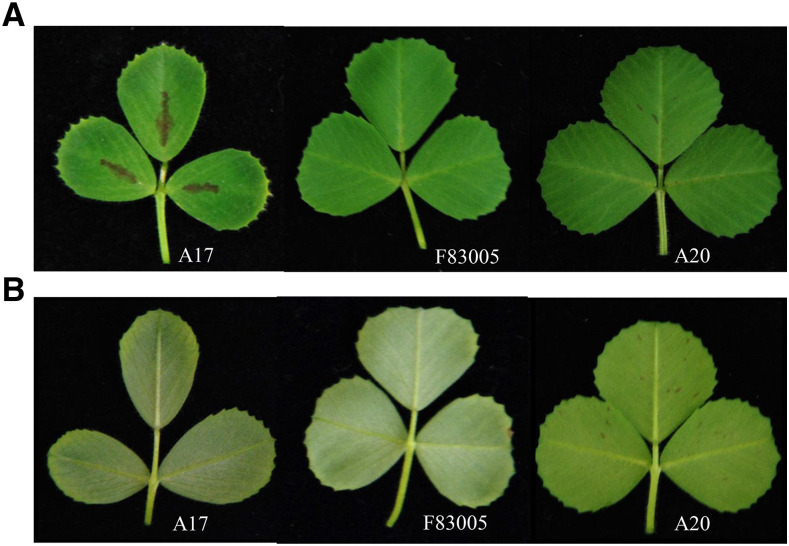
Pigmentation patterns on A17, F83005 and A20 leaves. A black or purple stain is displayed on the middle of adaxial leaf surface in A17, but not in F83005 and A20 (A). Leaf pigmentation is not shown on the abaxial surface in these ecotypes (B).

Three weeks after germination, black or purple pigmentation was observed at the adaxial midvein in A17 plants, but not on the abaxial surface (Figure 1). The leaf marking is absent on both upper and lower leaf sides in F83005 plants, whereas A20 leaves display infrequent and randomly distributed flecks (Figure 1). Of the 129 RILs of A17 × A20, a total of 59 individuals displayed the leaf marking caused by anthocyanin accumulation. Consistent with previous results (Thoquet *et al.* 2002), the segregation ratio fits 1:1 for presence to absence of the leaf marking (χ^2^ = 1.1, *df* = 1, *P* = 0.29), indicating that the lack of anthocyanin accumulation on leaves is controlled by a single dominant gene (Table 2). The single-gene pattern was also evidenced by the segregation ratio of 3:1 within F_2_ populations (Table 2). This gene was named *LPP1* (*Leaf Pigmentation Pattern 1*).

**Table 2:** Segregation ratio and Chi-square test analysis of RILs and F_2_s

Segregating population	Number of plants with leaf pigmentation	Number of plants without leaf pigmentation	Expected segregation ratio	Chi-square (χ^2^)	P-value
RILs of A17 x A20	59	70	1:01	0.938	0.33
F_2_ of A17 x A20	76	193	1:03	1.518	0.22
F_2_ of A17 x F83005	45	158	1:03	0.869	0.35

**Figure 2:**
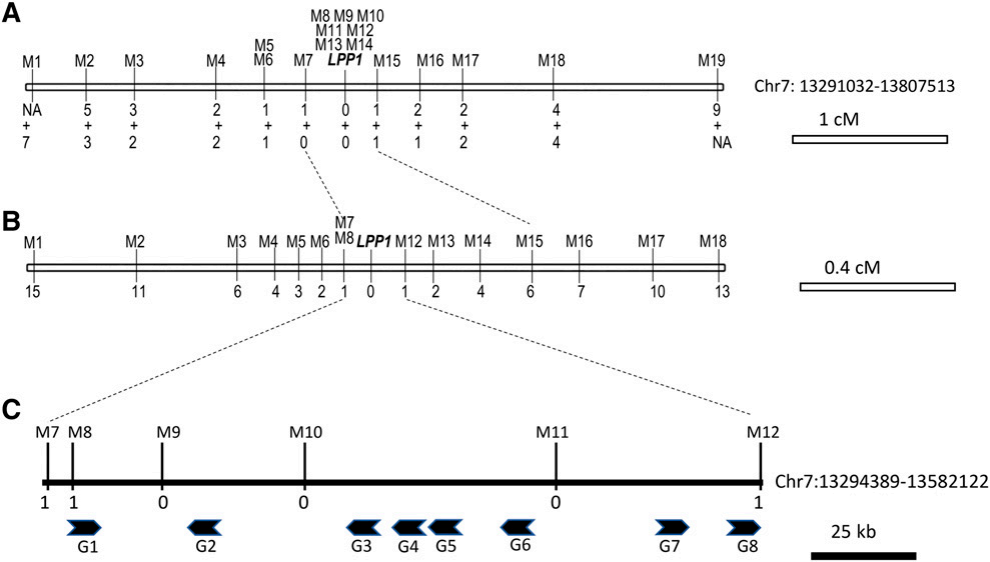
Genetic and physical mapping of the *LPP1* locus. A. Integrated genetic map generated with F_2_ populations derived and A17 × A20. *LPP1* is located on the *M. truncatula* molecular linkage group 7 (as indicated by the hollow box). Numbers indicate the number of recombination breakpoints separating the marker from *LPP1*, with the top and the bottom numbers are for the A17 × F83005 and A17 × A20 populations, respectively. B. Genetic map generated with RILs of A17 × A20. The genetic region of *LPP1* was narrowed by M8 and M12. Number of recombinant events were also indicated under markers. C. Physical map of the *LPP1* locus. Total of 8 protein-coding genes were identified in *LPP1* region. The maps are drawn to scale.

The F_2_ populations of A17 × A20 and A17 × F83005 were used first for genetic mapping of the *LPP1* gene. The same populations were also employed to localize the *SPC* (*Sense of Pod Coiling*) gene controlling pod coiling direction in *M. truncatula* (Yu *et al.* 2020). The SPC gene was anchored onto Chromosome 7 (Chr 7), and we found the phenotype markers of leaf pigmentation patten and pod coiling direction were closely linked (Yu *et al.* 2020). Therefore, we speculated that *LPP1* localized on the same chromosome with the *SPC* gene. Genetic mapping with CAPS markers confirmed localization of LPP1 on Chr 7 (Figure 2A). Flanked by the Marker 7 (M7) and M15, *LPP1* was delimited within a 516 kb-region (Chr 7:13291032-13807513). Assisted by RILs derived from A17 × A20, the *LPP1* region was narrowed down to 287 kb bordered by M8 and M12 (Chr 7: 13294389-13582122) (Figure 2B and 2C). All the markers harbored in this 287 kb-region, such as M9, M10, and M11, are co-segregating with leaf pigmentation phenotypes (Figure 2C).

Based on the RNA-seq data and EST alignments provided by the *M. truncatula* genome database (V4.2) (Young *et al.* 2011; Tang *et al.* 2014), we identified a total of 8 protein-coding genes within the 287 kb-region in the reference genome (Figure 2C, Table 3). Of those, three (G2, G4 and G6) are predicted to encode MYB transcription factors (Table 3). As for the other genes, their coding products are homologous to glutaredoxin C4 (G2), putative transmembrane protein (G3), peptide deformylase 1 (G5), polygalacturonase (G7), and C3HC4-type RING zinc finger protein (G8). The result of gene prediction was also confirmed by the newly released *M. truncatula* genome assembly V5.0 (MtrunA17Chr7: 13545781-13807677) (Pecrix *et al.* 2018).

**Table 3:** Predicated genes in the *LPP1* region

Gene number	Gene name	Predicted gene product
G1	Medtr7g035075	MYB transcription factor
G2	Medtr7g035245	Glutaredoxin C4
G3	Medtr7g035290	Transmembrane protein, putative
G4	Medtr7g035300	MYB-like DNA-binding domain protein
G5	Medtr7g035310	Peptide deformylase 1A
G6	Medtr7g035350	MYB transcription factor
G7	Medtr7g035415	Polygalacturonase
G8	Medtr7g035445	C3HC4-type RING zinc finger protein

In view of anthocyanin accumulation in A17 leaves being a recessive trait, lack of leaf pigmentation in A20 and F83005 may be caused by enzymatic degradation of anthocyanins or active suppression of their biosynthesis. Three candidate enzyme families have been proposed in anthocyanin degradation: polyphenol oxidase, peroxidase and β-glucosidases (Oren-Shamir 2009). Enzymatic in planta degradation of anthocyanins have been substantiated in Solanaceae and other families. Anthocyanin can be directly oxidized and degraded by peroxidase (Zipor *et al.* 2015). The other pathway is a two-step process, comprising deglycosylation by β -glucosidase and oxidation by polyphenol oxidase or peroxidase (Oren-Shamir 2009; Liu *et al.* 2018; Barbagallo *et al.* 2007). All these enzyme family members are missing in the *LPP1* genomic region.

Although some MYB transcription factors positively activate anthocyanin biosynthesis through the MBW complex, some are repressors that limit expression of the anthocyanin biosynthesis genes (Verdier *et al.* 2012; Liu *et al.* 2014; Peel *et al.* 2009). A series of R3- and R2R3-MYB repressors have been identified in *Arabidopsis* and other plants. Overexpression of *AtMYBL2* repressed anthocyanin biosynthesis in *Arabidopsis*, and knocking out *AtMYBL2* resulted in enhanced accumulation of anthocyanin (Matsui *et al.* 2008). Ectopic expression of *AtMYB60* in lettuce inhibited anthocyanin accumulation as well (Park *et al.* 2008). In grape, VvMYBC2-L3, VvMYB4b, VvMYB4a, and VvMYB4-like down-regulated the structural genes involved in flavonoid biosynthesis and reduced both PA and anthocyanin levels (Cavallini *et al.* 2015; Pérez-Díaz *et al.* 2016). RNAi-mediated silencing of *FcMYB1*, an MYB repressor gene in strawberry, led to increased accumulation of anthocyanins (Salvatierra *et al.* 2013). In *Medicago truncatula*, an R2R3-MYB protein, MtMYB2, was discovered as a transcriptional repressor in the regulation of both anthocyanin and PA biosynthesis (Jun *et al.* 2015).

Given that MYB transcription factor can be negative regulators of anthocyanin biosynthesis, three MYB genes in the *LPP1* region, G1, G4, and G6, were selected for sequence analysis. DNA sequencing did not identify any stop codons in the open reading frames of all three candidates in A17, A20 and F83005 (Table S3, S4, and S5). It is noteworthy that the allelic products of G1 in A17 and A20 share identical amino acid sequences, although their cDNAs vary with 2 single bp-substitutions (Table S3). It suggested that G1 may not be a candidate for the *LPP1* gene. Phylogenetic analysis indicated that for G4 and G6 allelic products of A20 and F83005 are more closely related to each other than to that of A17 (Figure S1). Moreover, G4 and G6 putatively encode R2R3-MYB transcription factors (Li *et al.* 2019). Therefore, G4 and G6 may be the *LPP1* candidates.

**Figure 3:**
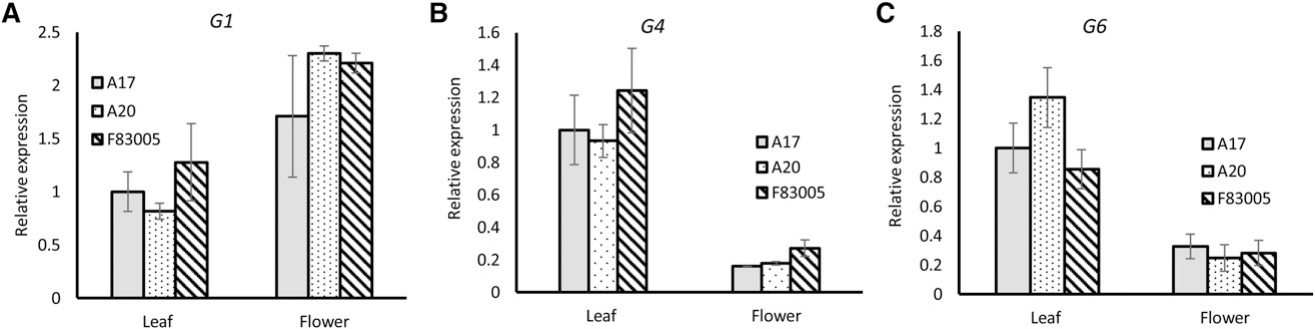
qRT-PCR analysis of gene expression levels for G1/*Medtr7g035075* (A) and G4/*Medtr7g035300* (B) and G6/*Medtr7g035350* (C) in leaf, open flower. The *actin* gene was used as internal control. The error bars indicate the standard errors (SEs).

To further strengthen their candidacy for these MYB transcription factor-coding genes, we conducted qRT-PCR analysis to characterize their expression profiles (Figure 3). Although leaf pigmentation is missing, anthocyanin accumulate in A20 and F83005 flowers to attract pollinators (Mouradov and Spangenberg 2014). We anticipate that the *LPP1* gene expresses highly in leaf but not in flower. As for G1, similar expression in leaf (Figure 3A) and upregulation in flower for three alleles, in combination with same allelic products, may exclude G1 as one of the *LPP1* candidates. On the contrary, both G4 (Figure 3B) and G6 (Figure 3C) were downregulated in flower, even though they highly express in leaf of all the three ecotypes. Thus, G4 and G6 were selected as strong candidates of *LPP1*. It is noteworthy that G4 and G6 are not differentially expressed in the leaf among genotypes (Figure 3). Therefore, the phenotypic difference between A17 and F83005/A20 may be caused by the protein sequence polymorphisms in the allelic products of G4 and G6 (Tables S4 and S5).

**Figure 4:**
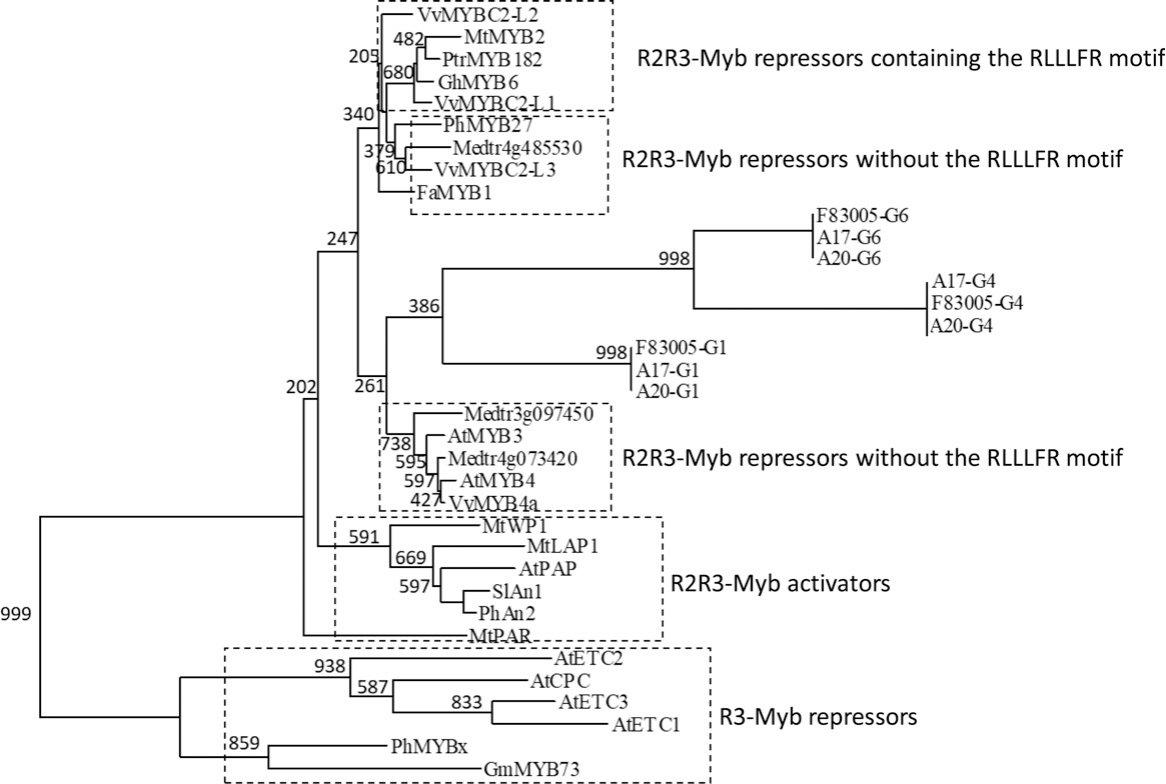
Phylogenetic tree based on a Neighbor-Joining (NJ) analysis of known MYB proteins with 1000 bootstrap pseudoreplicates. The tree was built using sequences from 6 R3-Myb repressors, 6 R2R3-Myb activators, 14 R2R3-Myb suppressors, and three allelic products of G1, G4 and G6. Protein sequences are given in Table S2. Branches with support of 200 or more are indicated. Values shown above the branches are the estimated amino acid substitutions per site (bar = 0.05).

MYB proteins directly or indirectly bind to *cis*-regulatory sequences of DNA to activate or inhibit gene expression, and conserved MYB-recognition elements are widely distributed throughout plant genomes (Hartmann *et al.* 2005). Suppression of transcription by R2R3-MYB repressors is achieved through a repression motif (TLLLFR) in their C termini (Matsui *et al.* 2008; Albert *et al.* 2014). AtMYBL2 or MtMYB2 competes with MYB-activators and forms suppressive MBW complex, and thus the suppression motif in these R2R3-MYB repressors inhibit expression of structural genes involved in anthocyanin biosynthesis (Matsui *et al.* 2008; Jun *et al.* 2015). Domain structure analysis based on the sequences of functionally investigated R3-/R2R3-Myb repressors and R2R3-Myb activators from various species indicated that the TLLLFR motif is missing in G1, G2 and G6 (Figure S2). However, this motif was only identified in 5 of the 16 R2R3-Myb repressors, suggesting that the TLLLFR motif may not be necessary for the suppression activity. A phylogenetic analysis was conducted to evaluate if a suppressive activity is associated with the R2R3-Myb proteins identified in the *LPP1* region (Figure 4). Although G1, G4 and G6 were grouped in an independent subclade, they were phylogenetically related to the R2R3-Myb repressors lacking the TLLLFR motif (Figure 4).

The anthocyanin biosynthesis pathway is completely present in A20 and F83005 plants, demonstrated by the normal color of the seed coat and flower. Therefore, spatiotemporal expression of MYB repressors in A20 and F83005 leaves is finely tuned, which is in line with the expression profile of candidate genes (Figure 3). Identification of the *LPP1* gene will further our understanding of the regulatory mechanisms underlying anthocyanin biosynthesis, and it may provide new perspective for the enrichment of vegetable, fruit, and forage with increased anthocyanins. Nevertheless, the identity of *LPP1* will be confirmed with genetic transformation or CRISPR/Cas9-mediated mutagenesis in A17 and A20, respectively.
